# Research on Control Strategy of Stainless Steel Diamond Plate Pattern Height Rolling Based on Local Constraints

**DOI:** 10.3390/ma18051116

**Published:** 2025-03-01

**Authors:** Zezhou Xin, Siyuan Qiu, Chunliu Wang, Huadong Qiu, Chuanmeng Sun, Zhibo Wu

**Affiliations:** 1 School of Electrical and Control Engineering, North University of China, Taiyuan 030051, China; xzz123202206@163.com (Z.X.); suncm@nuc.edu.cn (C.S.); 2 Olin College, Northeast Forestry University, Harbin 150040, China; qsy_sxty@163.com; 3 School of Mathematics, North University of China, Taiyuan 030051, China; chunliu.wang320@gmail.com; 4 TiSCO Co., Ltd., China Baowu Group, Taiyuan 030003, China; qhd_sxty@163.com

**Keywords:** local constraint, load distribution optimization, adaptive dynamic adjustment, MOARI-LC, stainless steel diamond plate, dimensional accuracy of plate shape, pattern height control

## Abstract

The rolling system for stainless steel, particularly in the production of diamond plates, represents a complex industrial control scenario. The process requires precise load distribution to effectively manage pattern height, due to the high strength, hardness, and required dimensional accuracy of the material. This paper addresses the limitations of offline methods, which include heavy reliance on initial conditions, intricate parameter settings, susceptibility to local optima, and suboptimal performance under stringent constraints. A Multi-Objective Adaptive Rolling Iteration method that incorporates local constraints (MOARI-LC) is proposed. The MOARI-LC method simplifies the complex multi-dimensional nonlinear constrained optimization problem of load distribution, into a one-dimensional multi-stage optimization problem without explicit constraints. This simplification is achieved through a single variable cycle iteration involving reduction rate and rolling equipment selection. The rolling results of HBD-SUS304 show that the pattern height to thickness ratio obtained by MOARI-LC is 0.20–0.22, which is within a specific range of dimensional accuracy. It outperforms the other two existing methods, FCRA-NC and DCRA-GC, with results of 0.19~0.24 and 0.15~0.25, respectively. MOARI-LC has increased the qualification rate of test products by more than 25%, and it has also been applied to the other six industrial production experiments. The results show that MOARI-LC can control the absolute value of the rolling force prediction error of the downstream stands of the hot strip finishing rolls within 5%, and the absolute value of the finished stand within 3%. These results validate the scalability and accuracy of MOARI-LC.

## 1. Introduction

Stainless steel diamond plate, characterized by a patterned surface of raised diamonds or lentils, is extensively utilized across the fields of industrial manufacturing, transportation, and construction engineering. Noted for its high strength and hardness [1,2], the rolling force required for this material is 30% to 100% greater than that for carbon steel of equivalent specifications. This increased rolling force escalates the load on rolling units and accelerates wear on the rolls. Additionally, the substantial deformation resistance and the resulting deformation rebound significantly impact the dimensional accuracy and service reliability of the product, necessitating enhanced rolling stability and load distribution [3,4]. Load distribution, a pivotal element of the rolling schedule, critically affects production stability, equipment wear, and product quality. Currently, the predominant methods for optimizing load distribution are manual adjustments based on experience and optimizations via intelligent algorithms.

In the realm of manual adjustment and mechanism research, Li et al. [5] developed an online numerical solution for calculating rolling force based on a friction model. Tao et al. [6] introduced a shape control strategy leveraging the negative movement of intermediate rolls. Furthermore, Gao et al. [7] elaborated a design method derived from the quadratic relationship between strain and coordinates. These advancements have yielded favorable outcomes in load distribution for most standard plate shapes. However, challenges persist with diamond plates due to their thickness specifications ranging from 1.8 to 8.0 mm, necessitating varied diamond roll reduction rates across different thicknesses. Manual adjustments to rolling force distribution often lead to the overflow of scrap steel when excessive rolling forces are applied to the upstream frame. Additionally, frequent changes in the rolling reduction rate at the diamond stand, prompted by manual adjustments, result in waviness and strip warping due to thickness fluctuations, compromising the accuracy and stability of the pattern height on the diamond plate. Owing to these deficiencies, numerous scholars have explored the optimization of load distribution using intelligent algorithms.

Gao et al. [8] aimed to suppress vibration and improve speed and used pattern search method for optimization. Peng et al. [9] used genetic algorithms to comprehensively consider heating energy consumption and rolling energy consumption to develop rolling schedules. Wang et al. [10] and Babajamali et al. [11] studied multi-objective optimization strategies for rolling schedules based on the NSGA-II algorithm, providing multiple solutions for rolling optimization in a single run of the algorithm, which has better adaptability and is superior to the initial empirical solution. Song et al. [12] proposed a collaborative control strategy for downstream stands in the SFR process using multi-objective NSGA-II algorithm, based on the wear characteristics and principles of work rolls. Li et al. [13] established a robust multi-objective optimization model for rolling plans and proposed a differential evolution algorithm based on evolutionary direction, which improved the profile compared to the actual rolling plan. Zhong et al. [14] proposed an adaptive differential evolution algorithm based on material forming mechanism and Bayesian optimization, which can effectively achieve global optimal solution. Li et al. [15] developed a multi-objective differential evolution algorithm to solve a mixed programming model with two objectives, and simulation results showed that it was superior to traditional methods used in practice. These studies have enhanced the rationality of rolling schemes, thereby improving product quality and boosting production efficiency. However, the efficacy of intelligent algorithms varies with problem characteristics, revealing some constraints when applied to the problems faced in this article: (1) multi-objective or discrete optimization problems involve complex parameter settings, where improper configurations can degrade algorithm performance; (2) poor adaptability to diverse steel grades and rolling conditions hampers the discovery of optimal strategies during dynamic changes; (3) the thin specification of stainless steel diamond plates, combining the rolling challenges of both stainless steel and diamond plates, presents significant dimensional accuracy issues, particularly in controlling pattern height, resulting in suboptimal application outcomes.

The current mechanism experience adjustment and intelligent algorithm optimization are basically carried out in offline environments, so the analysis results usually have a certain delay. This delay may result in loss of timeliness in scenarios that require immediate response and real-time processing, and requires pre-collection and processing of data, which can consume a significant amount of storage and processing resources. Offline methods for processing large amounts of data may require multiple iterations and optimizations, and accuracy may be affected to some extent when dealing with large amounts of data or complex processing procedures. Therefore, in response to the shortcomings of existing methods, this paper proposes a Multi-Objective Adaptive Rolling Iteration method based on local constraints (MOARI-LC). This method aims to transform the special multidimensional nonlinear optimization problem of load distribution into a one-dimensional multi-stage optimization problem without explicit constraints, and then decompose it into a series of sub problems for processing. By dynamically adjusting and optimizing the load distribution of the hot strip finishing rolls, the dimensional accuracy of the stainless steel diamond plate is improved, especially the accurate control of the pattern height, thus achieving a multi-objective optimization process for the load distribution of the hot strip finishing rolls.

## 2. Research Problem Analysis

### 2.1. Research Object and Existing Technology

Steel is the fundamental raw material for ensuring economic development and major engineering construction, among which hot strip rolling is the core process of steel production. Hot strip rolling is an intricate process involving mechanical, electrical, and hydraulic systems, characterized by multi-level, nonlinear, multi-variable, and strong coupling dynamics. As the diversity of products and specifications continues to grow, hot strip rolling has shifted towards a flexible production model marked by large spans, rapid cycles, high frequency, and elevated precision. Faced with the challenges of producing multiple varieties in a complex and dynamic environment, traditional single-feature modeling strategies fall short in meeting the precision requirements under conditions that involve significant changes in variety, specifications, and drafts of edger. In the hot strip rolling process, an effective distribution of rolling load maximizes equipment capacity, ensures product dimensional accuracy and internal quality, and enhances the stability of the rolling process [16]. With the development of artificial intelligence technology and new generation information technology, “Steel Industry 4.0” relies on technologies such as industrial big data and machine learning, to establish a new hot strip rolling model and intelligent control system. While implementing the development of primary control programs and secondary model system technologies, production adaptability applications are carried out to improve product quality, reduce production costs, and minimize energy consumption and pollution [17,18,19].

**Figure 1:**
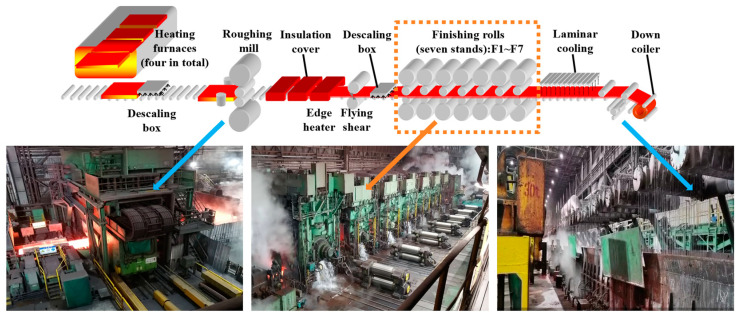
Layout of hot strip rolling process equipment.

As depicted in Figure 1, the primary stages of the hot strip rolling process include heating, descaling, rough rolling, finishing rolling, cooling, and coiling. Initially, slabs are transferred directly from the continuous casting machine in the steelmaking continuous casting workshop to the slab warehouse of the hot rolling workshop. According to the rolling schedule, slabs are then lifted by a crane to the loading roller of the heating furnace and subjected to a stepped heating process. Upon exiting the furnace, the heated slabs undergo descaling using a high-pressure water descaling device. These descaled slabs proceed to the roughing mill, where they are rolled into intermediate slabs of specified thickness. After the head and tail are trimmed, these intermediate billets are transferred to the finishing mill group. Here, through multi-pass rolling, they are transformed into finished strip steel that meets the desired specifications. Subsequently, the finished strips are cooled by a laminar cooling system, which reduces the temperature of the hot rolled strips from the final rolling temperature to the designated coiling temperature. Finally, the cooled strips are coiled into steel coils by the coiler, which adjusts parameters such as thickness and width during the winding process to ensure the quality of the steel coils.

In the production process of hot strip rolling, the finishing rolling stage is crucial for strip deformation and significantly influences the dimensional accuracy of the product’s plate shape [20,21]. Shape defects can severely compromise the quality of hot rolled strip steel [22]. To ensure optimal shape quality, it is essential to innovatively optimize both the distribution model and the control strategy [23,24,25]. Currently, the control system for hot strip rolling employs a dual-level computer control approach: process control via the Level 2 (L2) computer and basic automation through the Level 1 (L1) computer. The procedure for rolling force control is outlined as follows: calculate the rolling force using the L2 computer, transmit the calculation results to the L1 computer’s SDH module (Setting Agent Module), establish a communication channel between the SDH module and the rolling force control module, read the message data values, and send them to the drive system control block for the implementation and control of specific parameters.

### 2.2. Control Difficulties and Constraints

**Figure 2:**
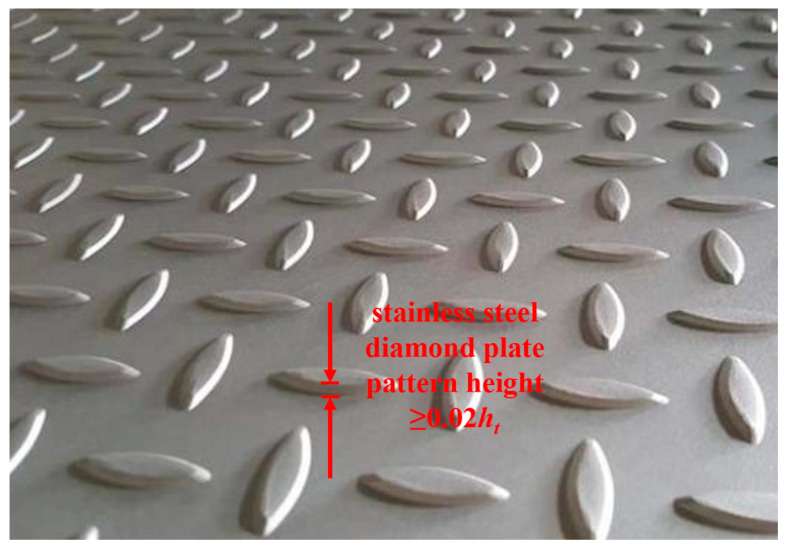
Height signal of the stainless steel diamond plate pattern.

The optimization model and control strategy of finishing rolls are central to the operation and a critical technology within the hot strip rolling production line. The primary challenges in rolling stainless steel diamond plate involve maintaining rolling stability and controlling the bloom rate. Thus, optimizing the load distribution across each frame, matching roll profiles, and refining control strategies are essential to ensure dimensional accuracy. In applications involving stainless steel diamond plate, the height of the diamond pattern is crucial for performance efficacy. This pattern height, defined as the elevation of the diamond above the steel plate surface, is illustrated in Figure 2. Insufficient pattern height compromises the anti-slip feature, whereas excessive height results in significant material loss during rolling. Consequently, the standard requirements for diamond plate pattern height stipulate that it must be at least 0.2 times the thickness of the base material. Currently, the technical challenges associated with rolling stainless steel diamond plate manifest primarily in two aspects: firstly, 20% of finished patterns exhibit a height less than the 0.2 times threshold; secondly, pattern height variability within the same batch can vary from 0.25 times to below 0.15 times the substrate thickness. Therefore, maintaining the accuracy and stability of the pattern height to thickness ratio is a crucial dimensional accuracy metric; yet, it also imposes unique boundary constraints.

Due to variations in rolling temperature, speed, reduction rate, and rolling force, continuous adjustments are essential for the smooth execution of the rolling process. These adjustments must account for actual working conditions, equipment capabilities, and process limitations. Specific boundary constraints to consider in the rolling process include the following:

(1) Rolling Force Constraint: The rolling force P at each stand must comply with equipment specifications, maintaining 0 < P < Pmax, where Pmax is the maximum allowable rolling force of the stand, measured in kilonewtons (kN);
(2) Rolling Moment Constraint: The rolling moment M at each stand should also satisfy equipment requirements, with 0 < M < Mmax, where Mmax represents the maximum allowable rolling moment of the stand, measured in kilonewton-meters (kN·m);
(3) Power Constraint: Each stand’s motor power N must adhere to 0 < N < Nmax, with Nmax being the stand’s maximum allowable motor power, expressed in kilowatts (kW);
(4) Motor Current Constraint: The motor current I should meet 0 < I < Imax, where Imax is the maximum allowable current for the stand, in amperes (A);
(5) Exit Entry Thickness Constraint: The exit entry thickness h of each stand must fall within 0 < h < hmax, where hmax is the maximum allowable value of exit entry thickness, in millimeters (mm).

Additionally, to meet the quality requirements of the final product, other constraints, such as maintaining the optimal shape of the finished plate, should also be considered.

## 3. Multi-Objective Adaptive Rolling Iteration Method Based on Local Constraints

### 3.1. Equipment Selection

**Figure 3:**
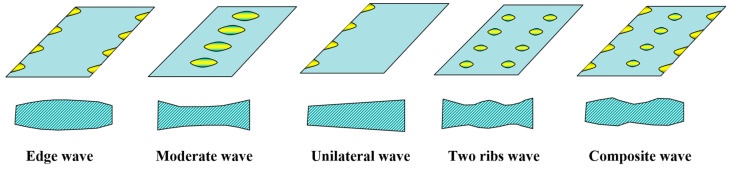
Strip warping wave shape.

In actual production, load distribution presents a complex challenge, as it entails balancing numerous interrelated factors within a multi-objective optimization framework. This complexity arises from the inherent conflicts among different optimization goals. A detailed analysis of existing methodologies reveals that a key factor contributing to instability is the fluctuation or inaccurate control of the reduction rate at the diamond stand during hot strip rolling. Typically, the end stand in the finishing rolls, which employs a diamond-engraved work roller, is tasked with imprinting the diamond pattern onto the strip surface. This stand not only requires a higher reduction rate but also experiences significant vibration. The frequent adjustments to the rolling reduction rate necessitated by the use of a diamond roller lead to variations in sheet thickness. Consequently, these fluctuations manifest as wave-shaped warping in the strip, as depicted in Figure 3, compromising both the stability and accuracy of the pattern height on stainless steel diamond plates.

**Figure 4:**
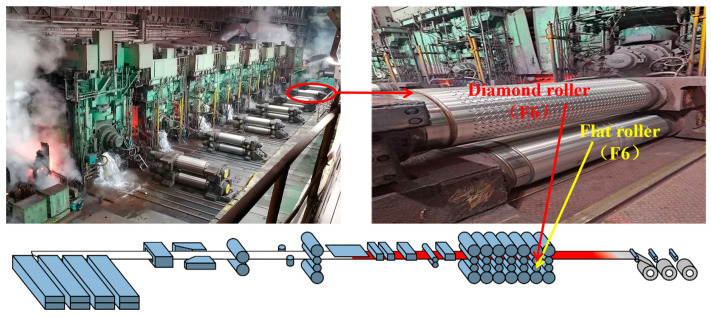
The diamond stand selection.

To leverage the positive impact of reduction rates and rolling forces on shape control, a Multi-Objective Adaptive Rolling Iteration method based on local constraints (MOARI-LC) has been proposed. This approach integrates objectives such as reasonable reduction distribution, balanced rolling force, and high dimensional accuracy of plate shape (the pattern height to thickness ratio ≥ 0.20). The focus of this paper is on the control of rolling force in finishing rolls, particularly improving the method of rolling force calculation using an L2 computer. The reduction rate at the diamond stand varies depending on the steel grade and specification; yet, it remains stable for identical steel grade specifications. Conversely, the reduction rates at other stands are dynamically adjusted. To maintain optimal dimensional accuracy of the product, the reduction rate at the end stand must be carefully moderated to ensure sufficient pattern height without being excessive. Consequently, as illustrated in Figure 4, the end stand F7 is omitted in this method. The work roller on the F6 stand utilizes a diamond roll, while the other rollers employ flat rolls. The reduction rate distribution across the remaining stands is dynamically and cyclically adjusted, utilizing a six-stand rolling process from F1 to F6.

### 3.2. Algorithm Step

After analyzing research challenges, establishing constraints, and completing equipment selection, the Multi-Objective Adaptive Rolling Iteration method was developed to enable online calculations. The core calculation of rolling force is given by the following formula:

(1) $$P_{ci}=coff_{1i}\times K_{i}\times \varepsilon_{i}$$

where *i* represents the stand number of the finishing rolls; *P_ci_* represents the calculated rolling force of stand *i*; *coff*_1*i*_ represents the rolling force correction factor of stand *i*; *K_i_* represents the Brinell hardness value of the steel, which can be tested according to the standard GB/T 231-2002; *ε_i_* represents the reduction rate of stand *i*.

**Figure 5:**
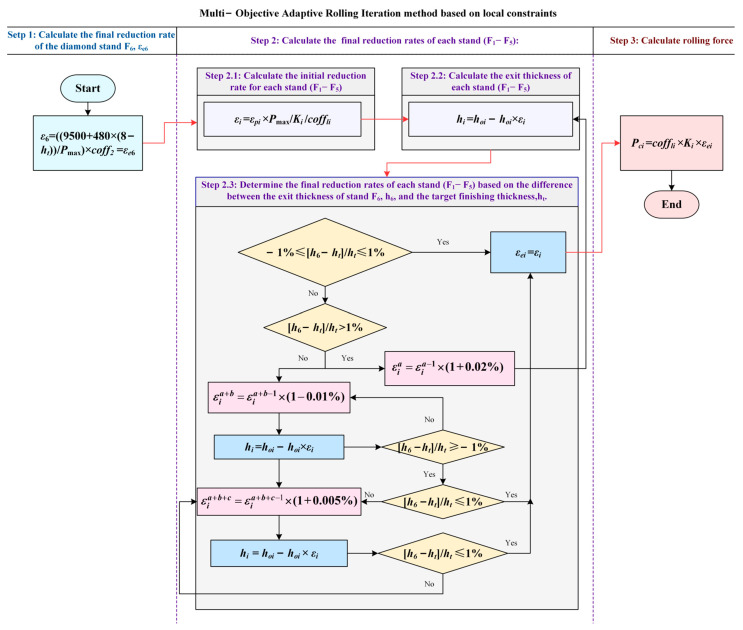
MOARI method flow chart.

The coff_1i_ calculation method is as follows:

(2) $$coff_{1i}=coff_{1(i-1)}+0.68\times (P_{i-1}-P_{c(i-1)})/P_{c(i-1)}$$

where *coff*_1(*i*−1)_ represents the rolling force correction factor of the previous stand, *P_i_*_−1_ represents the actual rolling force of the previous, and *P_c_*_(*i*−1)_ represents the calculated rolling force of the previous. The overall flow chart of MOARI method is shown in Figure 5:

Step 1: Calculate the final reduction rate *ε_e_*_6_ of the stand F6:

First, according to Formula (3), calculate the initial reduction rate *ε*_6_ of the stand F6:

(3) $$\varepsilon_{6}=\left\{\left[9500+480\times (8-h_{t})\right]/P_{max}\right\}\times coff_{2}$$

where *h_t_* represents the cooling value of the target thickness of finishing rolling; *P_max_* represents the maximum rolling force of each stand of the finishing rolls, taking 40,000 kN; *coff_2_* indicates the correction factor of stainless steel grade of the diamond plate, which is 0.73~0.83. The final reduction rate *ε_e_*_6_ of the diamond stand F6 is equal to the initial reduction rate *ε*_6_.

Step 2: The final reduction rate of the stand F1, F2, F3, F4, and F5, including the following steps:

(1) Step 2.1: Set the load plan downsizing distribution of the stand F1, F2, F3, F4, and F5 as *ε_p_*_1_, *ε_p_*_2_, ε*_p_*_3_, *ε_p_*_4_ and *ε_p_*_5_ respectively, and calculate the initial downsizing rate of each stand according to Formula (4):

(4) $$\varepsilon_{i}=\varepsilon_{pi}\times P_{max}/K_{i}/{coff}_{1i}$$

where *i* represents the stand number of the finishing rolls; *ε_i_* represents the initial reduction rate of the *i* stand; *P_max_* represents the maximum rolling force of each stand in finishing rolls; *K_i_* represents the hardness value of stand *i* steel; *coff*_1*i*_ represents the rolling force correction factor of stand *i*.

(2) Step 2.2: Calculate the exit thickness of the stand F1, F2, F3, F4, F5, and F6, according to Formula (5):

(5) $$h_{i}=h_{0i}-h_{0i}\times \varepsilon_{i}$$

where *i* represents the stand number of the finishing rolls; *h_0i_* represents the entry thickness of stand *i*. When *i* = 0, *h*_0*i*_ is the entry thickness *h_0_* of the finishing rolls; *h_i_* indicates the thickness of the exit of stand *i*, that is, the thickness of the exit of stand *i*, which is also the thickness of the entry of stand *i* + 1.

(3) Step 2.3: Determine the final reduction rate of the stand F1, F2, F3, F4, and F5 according to the difference [*h*_6_ − *h_t_*]/*h_t_* between the exit thickness *h*_6_ of stand F6 and the caloric value of the finishing thickness *h_t_*. According to the value of [*h*_6_ − *h_t_*]/*h_t_*, Step 2.3 can be implemented in three cases:

In the first case, when −1% ≤ [*h*_6_ − *h_t_*]/*h_t_* ≤ 1%*,* the final reduction rate of stand F1, F2, F3, F4, and F5 is equal to their respective initial reduction rate, that is, the final reduction rate *ε_ei_* = *ε_i_*, where *i* is 1, 2, 3, 4, or 5.

In the second case, when [*h*_6_ − *h_t_*]/*h_t_* > 1%, Step 2.3 includes the following steps:

① Repeat the calculation of *ε_i_^a^* according to formula *ε_i_^a^* = *ε_i_^a^^−^*^1^ × (1 + 0.02%), then calculate [*h*_6_ − *h_t_*]/*h_t_* from *ε_i_^a^* to [*h*_6_ − *h_t_*]/*h_t_* ≤ 1%; end the cycle calculation, and record the value of *ε_i_^a^*, where *a* is a positive integer representing the number of iterations, and *i* represents stand *i*. This means the following:

On the basis of the initial reduction rate of stand F1 to F5, each stand is increased by 0.02%; that is, according to *ε_i_*^1^ = *ε_i_* × (1 + 0.02%), to calculate *ε_i_*^1^, the value of stand *i* is 1, 2, 3, 4, or 5. Bring the calculated *ε_i_*^1^ into the Formula (5) above, recalculate *h*_6_ with *ε_i_*^1^ instead of *ε_i_*, and recalculate the value of [*h*_6_ − *h_t_*]/*h_t_*.

If the recalculated value of [*h*_6_ − *h_t_*]/*h_t_* is still greater than 1%*,* the cycle continues with the calculation of 0.02% increase in the reduction rate per stand for stands F1 to F5. That is, *ε_i_^2^* is calculated according to *ε_i_*^2^ = *ε_i_*^1^ × (1 + 0.02%), *h*_6_ is calculated with *ε_i_^2^* according to Formula (5) and the value of [*h*_6_ − *h_t_*]/*h_t_* is recalculated. Calculate *ε_i_^a^* according to *ε_i_^a^* = *ε_i_^a^^−^*^1^ × (1 + 0.02%), calculate *h*_6_ with *ε_i_^a^* according to Formula (5), and recalculate the value of [*h*_6_ − *h_t_*]/*h_t_* until *h*_6_ is recalculated with *ε_i_^a^*, according to Formula (5), to make [*h*_6_ − *h_t_*]/*h_t_* ≤ 1%; end the cycle calculation and record the value of *ε_i_^a^*.

② Determine whether [*h*_6_−*h_t_*]/*h_t_* is greater than or equal to −1% at the end of the cycle calculation. If yes, the final reduction rate of each stand *ε_ei_* = *ε_i_^a^*, and can enter Step 3. If no, that is, [*h*_6_ − *h_t_*]/*h_t_* < −1%*,* go to ③.

③ Repeat the calculation of *ε_i_^a+b^* according to formula *ε_i_^a+b^ = ε_i_^a+b^^−^*^1^ × (1 − 0.01%)*,* and then calculate the steps of [*h*_6_ − *h_t_*]/*h_t_* according to *ε_i_^a+b^* until [*h*_6_ − *h_t_*]/*h_t_* ≥ −1%; end the cycle calculation, and record the value of *ε_i_^a+b^*, where *b* is a positive integer representing the number of iterations, and *i* represents stand *i*. This means the following:

Cycle through the calculation of reducing the reduction rate of stands F1 to F5 by 0.01% per stand until [*h*_6_ − *h_t_*]/*h_t_* > −1% is satisfied, that is, *ε_i_^a+^*^1^ *= ε_i_^a^* × (1 − 0.01%); put the calculated *ε_i_^a+^*^1^ into the above Formula (5), replace *ε_i_* with *ε_i_^a+^*^1^ to recalculate *h*_6_, and recalculate the value of [*h*_6_ − *h_t_*]/*h_t_*.

If the recalculated value of [*h*_6_ − *h_t_*]/*h_t_* is still less than −1%, then continue to cycle through the calculation of 0.01% reduction in the drive rate per stand of stands F1 to F5; that is, calculate *ε_i_^a+2^* according to *ε_i_^a+2^ = ε_i_^a+^*^1^ × (1 − 0.01%)*,* bring *ε_i_^a+^*^2^ into Formula (5) to calculate *h*_6_, and recalculate the value of [*h*_6_ − *h_t_*]/*h_t_*. Calculate *ε_i_^a+b^* according to *ε_i_^a+b^ = ε_i_^a+b^^−^*^1^ × (1 − 0.01%), bring *ε_i_^a+b^* into Formula (5) to calculate *h*_6_, recalculate the value of [*h*_6_ − *h_t_*]/*h_t_*, until *ε_i_^a+b^* recalculates *h*_6_, according to Formula (5), to make [*h*_6_ − *h_t_*]/*h_t_* > −1%; end the cycle calculation, and record the value of *ε_i_^a+b^*.

④ Determine whether [*h*_6_ − *h_t_*]/*h_t_* is less than or equal to 1% at the end of the loop calculation: if so, the final reduction rate *ε_ei_* = *ε_i_^a+b^*, can be entered into step3; if no, that is, [*h*_6_ − *h_t_*]/*h_t_* > 1%, go to ⑤.

⑤ Repeat to calculate *ε_i_^a+b+c^* according to formula *ε_i_^a+b+c^ = ε_i_^a+b+c^^−^*^1^ × (1 + 0.005%), then calculate the steps of [*h*_6_ − *h_t_*]/*h_t_* according to *ε_i_^a+b+c^* until [*h*_6_ − *h_t_*]/*h_t_* ≤ 1%, end the loop calculation and record the value of *ε_i_^a+b+c^* as *ε_ei_*, where *c* is a positive integer representing the number of iterations, and *i* represents stand *i*.

This means the following: Cycle through the calculation of 0.005% increase in the reduction rate of stands F1 to F5 per stand until it meets [*h*_6_ − *h_t_*]/*h_t_* ≤ 1%. That is, *ε_i_^a+b+^*^1^ *= ε_i_^a+b^* × (1 + 0.005%) puts the calculated *ε_i_^a+b+^*^1^ into the above Formula (5), recalculate *h*_6_ with *ε_i_^a+b+^*^1^ instead of *ε_i_*, and recalculate the value of [*h*_6_ − *h_t_*]/*h_t_*:

If the recalculated value of [*h*_6_ − *h_t_*]/*h_t_* is still greater than 1%*,* then continue the cycle of stands F1 to F5 with a 0.005% increase in the reduction rate per stand. That is, calculate *ε_i_^a+b+2^* according to *ε_i_^a+b+2^ = ε_i_^a+b+^*^1^ × (1 + 0.005%)*,* use *ε_i_^a+b+2^* to calculate *h*_6_ according to Formula (5), and recalculate the value of [*h*_6_ − *h_t_*]/*h_t_*; Calculate *ε_i_^a+b+c^* according to *ε_i_^a+b+c^ = ε_i_^a+b+c^^–^*^1^ × (1 + 0.005%)*,* use *ε_i_^a+b+c^* to calculate *h*_6_ according to Formula (5), recalculate the value of [*h*_6_ − *h_t_*]/*h_t_*, the loop calculation ends when *ε_i_^a+b+c^* is used to recalculate *h*_6_ to [*h*_6_ − *h_t_*]/*h_t_* ≤ 1% according to Formula (5).

After a large number of data verification, the above steps can ensure − 1% ≤ [*h*_6_ − *h_t_*]/*h_t_* ≤ 1%. Therefore, the value *ε_i_^a+b+c^* at this time is recorded as the final reduction rate *ε_ei_*.

In the third case, when [*h*_6_ − *h_t_*]/*h_t_* < −1%*,* you can follow ③ through ⑤ in the second case above. Step 2.3 consists of the following steps:

⑥ Repeat the calculation of *ε_i_^x^* according to formula *ε_i_^x^ = ε_i_^x^^−^*^1^ × (1 − 0.01%)*,* and then calculate the steps of [*h*_6_ − *h_t_*]/*h_t_*, according to *ε_i_^x^* until [*h*_6_ − *h_t_*]/*h_t_* ≥ −1%, ending the cycle calculation. Record the value of *ε_i_^x^*, where *x* is a positive integer, representing the number of iterations, and *i* represents stand *i*;

⑦ Determine whether [*h*_6_ − *h_t_*]/*h_t_* is less than or equal to 1% at the end of the loop calculation: If so, the final reduction rate *ε_ei_* = *ε_i_^x^*; if no, go to ⑧.

⑧ Repeat to calculate *ε_i_^x+y^* according to formula *ε_i_^x+y^ = ε_i_^x+y^^−^*^1^ × (1 + 0.005%)*,* then calculate the steps of [*h*_6_ − *h_t_*]/*h_t_* according to *ε_i_^x+y^* until [*h*_6_ − *h_t_*]/*h_t_* ≤ 1%*,* end the cycle calculation, and record the value of *ε_i_^x+y^* as *ε_ei_* at this time, where *y* is a positive integer representing the number of iterations, and *i* represents stand *i*.

Step 3, The rolling force of each stand is calculated according to the final reduction rate of each stand:

Calculate rolling force according to Formula (6):

(6) $$P_{ci}=coff_{1i}\times K_{i}\times \varepsilon_{ei}$$

where *P_ci_* represents the calculated rolling force of stand *i; coff*_1*i*_ represents the rolling force correction factor of stand *i; K_i_* represents the hardness value of stand *i* steel; *ε_i_* indicates the final reduction rate of stand *i*.

## 4. Simulation Test and Analysis

### 4.1. Simulation Experiment

In this experiment, the Multi-Objective Adaptive Rolling Iteration (MOARI-LC) method was employed to optimize the load distribution across seven-stand hot strip finishing rolls. This optimization aimed to validate the effectiveness of the method in controlling the accuracy and stability of the pattern height on stainless steel diamond plates. The simulation utilized austenitic stainless steel HBD-SUS304, with steel coil number 993903701. The specific composition of the steel is detailed in Table 1.

Parameters were established based on actual conditions, including a target thickness of 4.88 mm, a target width of 1249 mm, a finishing rolling target thickness of 4.975 mm, an entry thickness for finishing rolling of 35.803 mm, and an entry width for finishing rolling of 1285.79 mm. The specific setting coefficients for each stand are presented in Table 2, and the simulation results for the reduction rate are detailed in Table 3.

**Figure 6:**
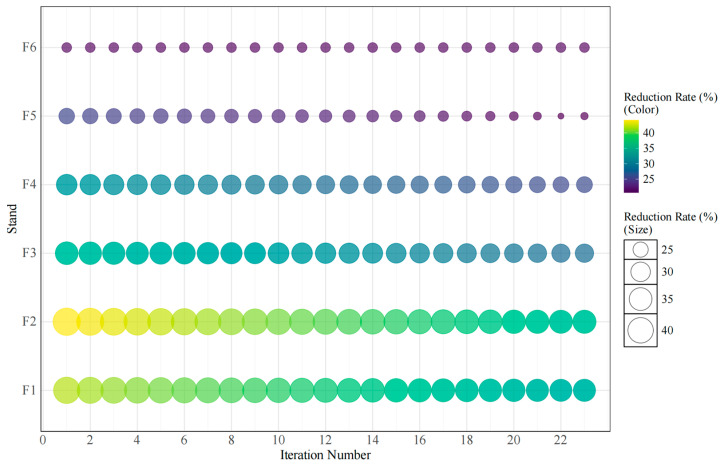
Iterative optimization of each stand reduction rate.

As illustrated in Figure 6, the iterative optimization of each stand’s reduction rate involves a process of deriving a series of approximate solutions from an initial value. This process is governed by identifying the iterative variables and establishing their relationships. The algorithm comprehensively accounts for process constraints by converting these constraints into mathematical expressions and integrating them into the optimization model. Moreover, the rigidity of these constraints is evaluated to fine-tune the progress of rolling iterative optimization. Continuous adaptive optimization and the updating of variable parameters, when combined with an online feedback correction mechanism, enhance optimization efficiency and fault tolerance. This results in improved real-time system stability amidst dynamic changes and external disturbances.

The industrial production experiment of stainless steel diamond plate demonstrates that the Multi-Objective Adaptive Rolling Iteration (MOARI-LC) method yields impressive results. With fewer iterations, the actual values quickly converge to the standard values, optimizing the reduction distribution at each stand more effectively and thus directly increasing product yield. By setting and adjusting three different asynchronous long control iteration directions and minimizing the number of iterations, the convergence of the function is significantly improved. This approach also ensures the stability and reliability of the iterative optimization of the reduction rate. The specific data regarding the load distribution results for each stand are presented in Table 4.

**Figure 7:**
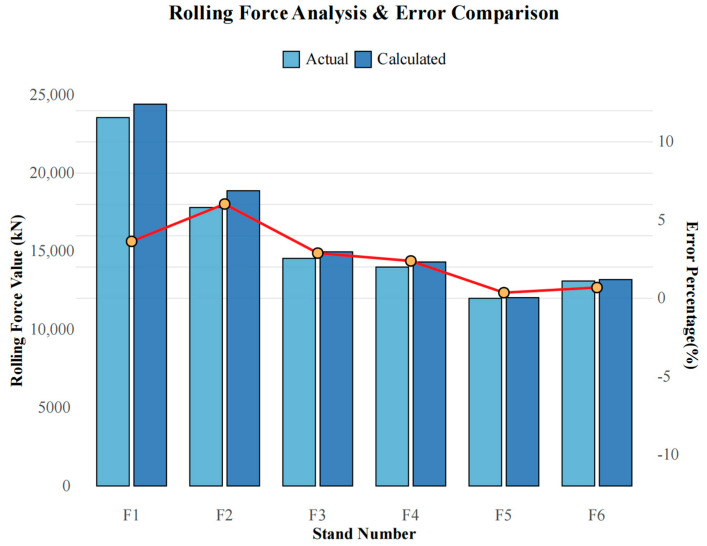
Predicted rolling force, actual rolling force, and percentage error of each stand.

The Multi-Objective Adaptive Rolling Iteration (MOARI-LC) method simplifies the complex multi-dimensional nonlinear constrained optimization problem of load distribution into a one-dimensional monotone optimization problem. This simplification is achieved through a single variable cycle iteration involving reduction rate and roll equipment selection. Consequently, multi-objective optimization is transformed into a single-objective optimization focused solely on the reduction rate. This allows the multi-stage problem to be decomposed into a series of sub-problems, each designed to achieve specific objectives and performance indicators. To validate the model’s accuracy, the predicted rolling force calculated by the model was compared with the actual rolling force measured onsite, as depicted in Figure 7. The results indicate a high degree of alignment between the calculated predictions and the actual measurements. Notably, except for stand F2, where the error is 6.03%, the errors in stands F1 to F6 are less than 5% and exhibit a decreasing trend. This demonstrates the optimization process’s convergence and the model’s capacity for real-time responsiveness.

Due to the large reduction amount and reduction rate of the upstream stands (F1, F2, F3), as well as the fluctuation of environmental factors such as rolling speed and temperature [26], it is normal for prediction errors to sometimes exceed 5%. In the rolling production process of diamond plates, the finished stand is F6, so it is necessary to control the prediction error of F6 as small as possible. When the prediction error of F2 exceeds 5% as shown in Figure 7, the impact on the finished stand F6 and the final diamond plate product can be reduced by adjusting the stand F3, F4, and F5. The MOARI-LC method utilizes the quantitative relationship between the reduction rate of each stand, rolling force, and load distribution to achieve multi-objective optimization of load distribution in hot strip finishing rolls. By adjusting the reduction rate of each stand to compensate for the uncertain effects caused by changes in rolling environment and conditions, it is beneficial to improve the rolling stability of hot strip finishing rolls and the dimensional accuracy of strip steel.

Calculate the correlation coefficient between every two indicators according to Formula (7), and draw a correlation coefficient heatmap. Based on the correlation coefficients corresponding to different block colors in the heat map, the magnitude of the correlation between indicators can be determined. Formula (7) is as follows:

(7) $$\rho_{X_{1}X_{2}}=\frac{Cov(X_{1},X_{2})}{\sqrt{DX_{1},DX_{2}}}=\frac{EX_{1}X_{2}-EX_{1}\times EX_{2}}{\sqrt{DX_{1}\times DX_{2}}}$$

**Figure 8:**
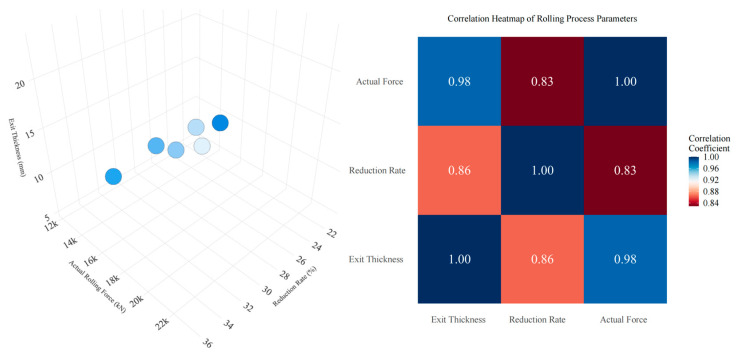
The spatial distribution and correlation of reduction rate, rolling force, and exit thickness.

Figure 8 illustrates the distribution and correlation degree of actual data in the target space. The three indices—reduction rate, rolling force, and exit thickness—are coupled to a certain extent; a change in any one index may induce changes in the other two. Thus, from a practical perspective, the adaptive rolling iterative optimization, which transforms multiple variables into single variables under different objective constraints, can effectively address the coupling issue. This approach helps to balance the potential correlation effects among various objectives. Exit thickness directly influences the plate shape, while reduction rate and rolling force impact the exit thickness. Therefore, ensuring a reasonable reduction distribution and a balanced rolling force are crucial for maintaining the dimensional accuracy of the plate. As evidenced by Figure 8, the correlation between exit thickness and rolling force, at 0.98, is stronger than that between the reduction rate, at 0.86. Consequently, a technical approach that first optimizes rolling force followed by the adjustment of exit thickness through the reduction rate is viable. Moreover, to control strip thickness effectively, the reduction rate should remain stable without frequent fluctuations, and the rolling force should be neither too high nor too low. These principles align with observations from actual production, confirming that the load allocation obtained by MOARI-LC is reasonable.

**Figure 9:**
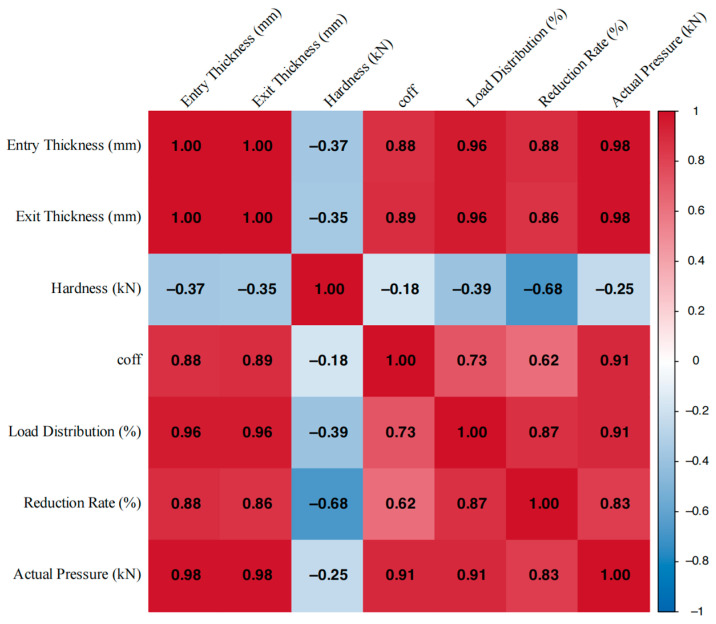
Load distribution index synthesis and correlation.

After ensuring the reasonable and stable thickness of the exit, in order to further ensure the dimensional accuracy of the plate shape, and effectively control the pattern height of the diamond plates. It is necessary to combine other production equipment and process technologies, and analyze the comprehensive correlation requirements of matching multiple indicators as shown in Figure 9. The correlation relationship between multiple indicators with distinctive features can be effectively characterized by a correlation heatmap. It represents the magnitude of the correlation coefficient between two indicators through the depth of colors, intuitively identifying significant positive or negative correlations between variables. In general, |*r*| ≤ 0.3 indicates low correlation, 0.3 < |*r*| ≤ 0.5 indicates moderate correlation, 0.5 < |*r*| ≤ 0.8 indicates strong correlation, and |*r*| > 0.8 is highly correlated. From Figure 9, it can be seen that hot strip rolling is a complex industrial process with multi-level, nonlinear, multivariable, and strong coupling characteristics. Multiple indicators in the rolling schedule have strong coupling relationships. The correlation between the reduction rate and other indicators is weak, so it is reasonable to use local constraints on the F6 stand through the selection of rolling equipment and iteratively obtain the optimal solution for the single variable reduction rate online. This transforms the special multidimensional nonlinear optimization problem of load distribution into a one-dimensional multi-stage optimization problem without explicit constraints. Then, decompose it into a series of sub problems to gradually achieve balanced rolling force and reasonable exit thickness, which is a sufficient condition for ensuring product quality and dimensional accuracy.

### 4.2. Validation Analysis

**Figure 10:**
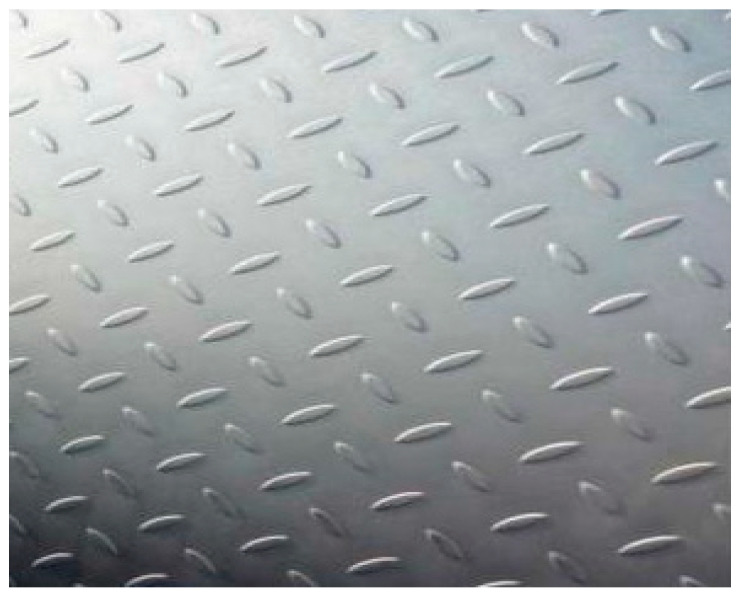
Austenitic stainless steel diamond plate HBD-SUS304 product appearance.

As depicted in Figure 10, following the rolling of austenitic stainless steel HBD-SUS304, the actual thickness of the measured diamond plate is 4.885 mm, with the deviation from the target thickness of 4.88 mm falling within acceptable limits. The measured pattern height is 0.991 mm, and the pattern height to thickness ratio is 0.203 (≥0.20), satisfying the standard requirements. This method effectively controls the reduction rate at a specific stand (F6), transcending the restrictive bounds of local constraints and maintaining stability in the reduction rate amidst rolling force fluctuations. Additionally, the rolling force remains stable through adaptive dynamic adjustment, despite fluctuations in the reduction rate from stands F1 to F5. This ensures that the pattern height of the stainless steel diamond plate remains within standard limits without compromising the strip’s exit plate shape or inducing medium and bilateral waves.

**Figure 11:**
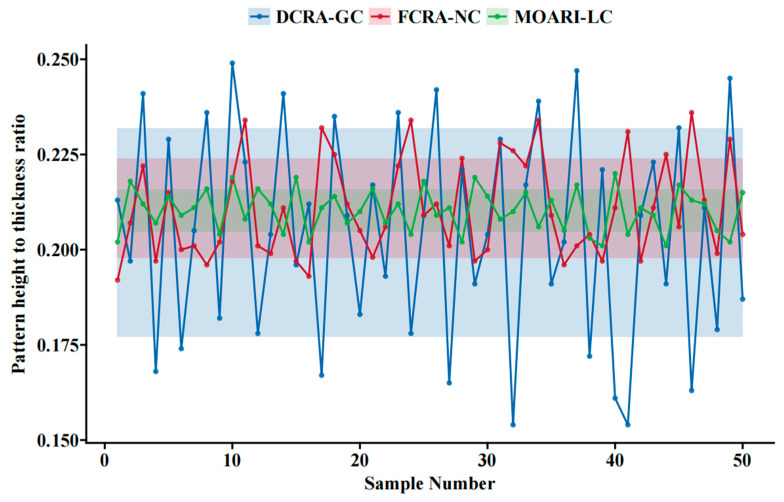
Statistics and variance distribution of the pattern height to thickness ratio data obtained by the three methods.

**Figure 12:**
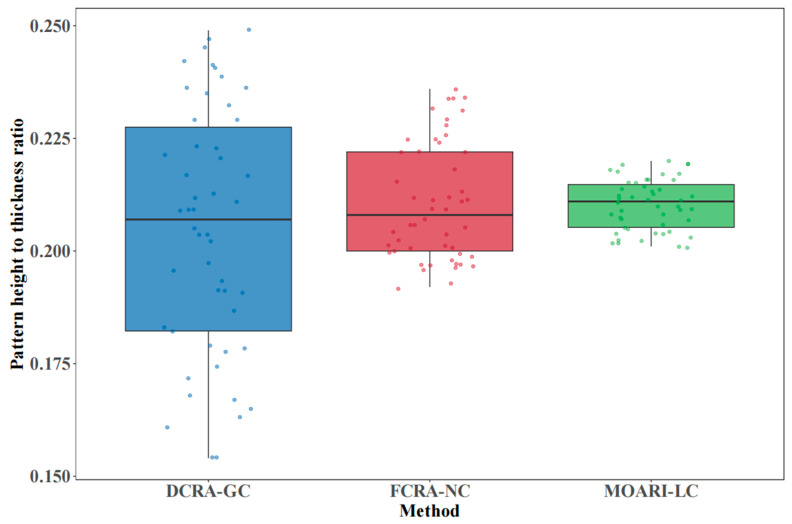
Data distribution of the pattern height to thickness ratio obtained by three methods.

By sampling the stainless steel diamond plates produced on a hot strip rolling line of an iron and steel enterprise, the pattern height to thickness ratios obtained by three methods were calculated. They include MOARI-LC, the fixed compression rate allocation method (no constraints), and the dynamic compression rate allocation method (global constraints). This article selects Mean Absolute Error (MAE), Root Mean Square Error (RMSE), and qualification rate as evaluating indicators to evaluate the experimental application effects of different methods. The calculation formula for evaluation indicators is as follows:

(8) $$MAE=\frac{1}{n}\sum_{i=1}^{n}\left|y_{i}-y_{i0}\right|$$

(9) $$RMSE=\sqrt{\frac{1}{n}\sum_{i=1}^{n}{\left(y_{i}-y_{i0}\right)}^{2}}$$

where *n* represents the number of samples; *y_i_* represents the true value of pattern height to thickness ratio for the sample; *y_i_*_0_ represents the standard value. The comparative effects of different methods are shown in Table 5:

The results shown in Figure 11 indicate that the MOARI-LC method can control the pattern height to thickness ratio of stainless steel diamond plates between 0.20 and 0.22. It outperforms the existing FCRA-NC and DCRA-GC methods with results of 0.19–0.24 and 0.15–0.25, respectively. This indicates that the optimization results consistently approach the standard target value and meet the dimensional accuracy requirements for plate shape, as further illustrated in Figure 12. The efficacy of the MOARI-LC method in meeting production demands through accurate calculation predictions and rolling stability has been substantiated. This method enhances equipment productivity, boosts efficiency, and reduces costs. Additionally, optimizing the steel grade correction coefficient (*coff*_2_) for different varieties can enhance the prediction accuracy of the model. Consequently, the proposed model is adaptable to the specific conditions of various production lines and capable of reflecting the differences in rolling environmental conditions. It is extensible and portable, making it readily applicable to the rolling processes of different scales and varieties.

### 4.3. Expand Applications

The rolling results of HBD-SUS304 on the industrial production line show that the pattern height to thickness ratio obtained by MOARI-LC is 0.20–0.22, which is within a specific range of dimensional accuracy. It outperforms the other two existing methods FCRA-NC and DCRA-GC with results of 0.19~0.24 and 0.15~0.25, respectively. MOARI-LC has increased the product qualification rate by over 25%, which is beneficial for ensuring production efficiency, reducing production costs, and minimizing material waste.

**Figure 13:**
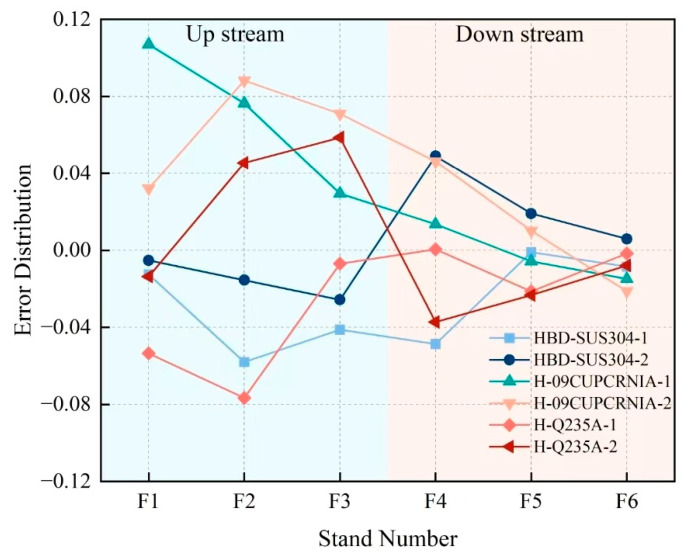
Distribution of rolling force prediction errors for each stand under 6 different conditions.

**Figure 14:**
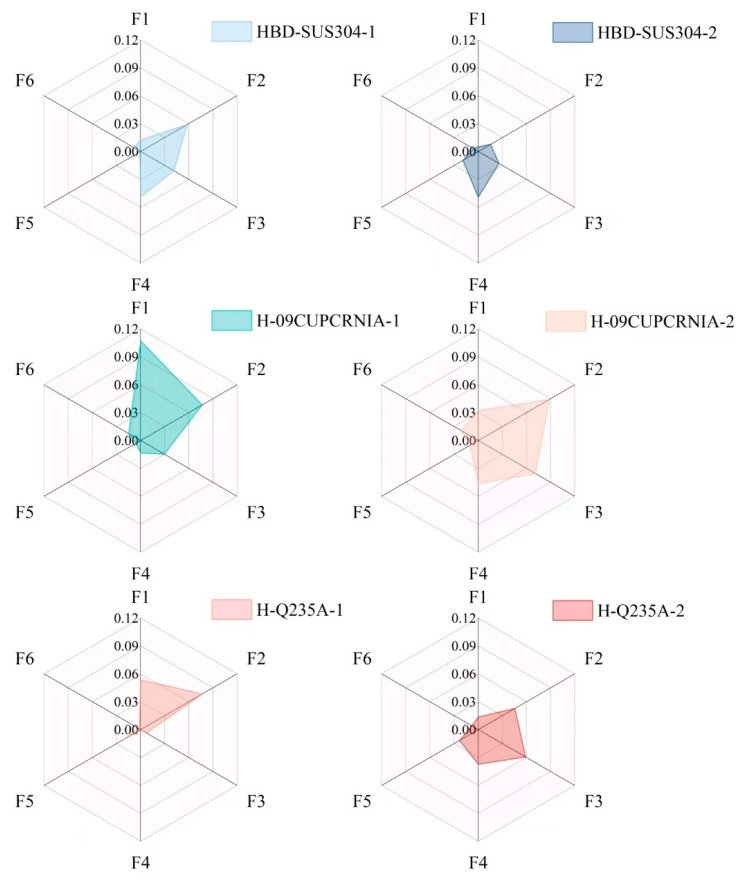
Comparison of prediction errors of rolling force between upstream and downstream stands under 6 different conditions.

We also applied MOARI-LC to industrial production experiments of two different sizes of stainless steels HBD-SUS304-1 (Type 2) and HBD-SUS304-2 (Type 3), two weather resistant steels H-09CUPCRNIA-1 (Type 4) and H-09CUPCRNIA-2 (Type 5), two plain carbon steels H-Q235A-1 (Type 6) and H-Q235A-2 (Type 7). The specific components of the six materials are shown in Table 6:

The results shown in Table 7 indicate that: MOARI-LC can control the absolute value of the rolling force prediction error of the downstream stands within 5%, and the absolute value of the finished stand within 3% in these 6 situations. As shown in Figure 13 and Figure 14, the upstream stands (F1, F2, F3) have a large reduction amount and a high reduction rate, and are greatly affected by environmental factors such as rolling speed and temperature fluctuations. Therefore, it is normal for prediction errors above 5% to occur at times. Due to the fact that in the rolling production process of diamond plates, it is necessary to control the prediction error of the finished stand F6 as small as possible. When the prediction error of the upstream stands exceeds 5%, the downstream stands can be adjusted to reduce the impact on the finished stand F6 and the final diamond plate product.

After obtaining smaller prediction errors for downstream stands, especially the finished stand, sensor calibration and real-time adjustment are completed. Rolling tests are conducted on stainless steels HBD-SUS304-1 (Type 2) and HBD-SUS304-2 (Type 3), weather resistant steels H-09CUPCRNIA-1 (Type 4) and H-09CUPCRNIA-2 (Type 5), plain carbon steels H-Q235A-1 (Type 6) and H-Q235A-2 (Type 7). After rolling, the measured pattern height to thickness ratios shown in Table 8 are 0.201, 0.203, 0.204, 0.204, 0.211, and 0.209, all of which are greater than 0.20 and still meet the dimensional accuracy requirements of the diamond plates. These results validate the scalability and accuracy of the MOARI-LC method, which can effectively unleash the potential of existing devices. It can improve the production efficiency of steel diamond plate related products and is of great significance for the stable transition of actual industrial production.

## 5. Conclusions

The load distribution model for hot strip finishing rolls is central to the hot strip production process, with reasonable load distribution playing a critical role in rolling speed, operational efficiency, product quality, and production cost. Given the high strength, hardness of stainless steel, and the required dimensional accuracy for diamond plates, the precision of the load distribution model impacts not only product quality but also the development of other products. In response to the shortcomings of existing methods, this paper proposes a Multi-Objective Adaptive Rolling Iteration method based on local constraints (MOARI-LC).

MOARI-LC transforms the special multidimensional nonlinear optimization problem of load distribution, into a one-dimensional multi-stage optimization problem without explicit constraints, through rolling equipment selection and single variable online loop iteration of reduction rate. Further decomposing it into a series of sub problems for processing has improved the dimensional accuracy of stainless steel diamond plates, especially the accurate control of pattern height, ultimately achieving multi-objective optimization of load distribution. This article provides a detailed introduction to the initial compression allocation, compression calculation for each stand, adjustment strategy process of the proposed method, and further analyzes the necessity of applying multi-objective constraint transformation strategies. During the rolling process of stainless steel diamond plates, simulation calculations and HBD-SUS304 industrial experiments have demonstrated the feasibility and effectiveness of the proposed MOARI-LC method. It can control the pattern height to thickness ratio of stainless steel diamond plates between 0.20 and 0.22, which is better than the results obtained by FCRA-NC method and DCRA-GC method of 0.19~0.24 and 0.15~0.25.

After load optimization using the MOARI-LC method, the dimensional accuracy of the stainless steel diamond plate is closer to the target value, increasing the qualification rate of the test products by more than 25%. This is beneficial for ensuring production efficiency, reducing production costs, and minimizing material waste. Subsequently MOARI-LC is applied to the industrial production experiments, including two different sizes of stainless steel HBD-SUS304-1 and HBD-SUS304-2, two types of weather resistant steel H-09CUPCRNIA-1 and H-09CUPCRNIA-2, two types of plain carbon steel H-Q235A-1 and H-Q235A-2. The results show that MOARI-LC can control the absolute value of the rolling force prediction error of the downstream stands within 5%, and the absolute value of the finished stand within 3% in the other 6 cases, and the dimensional accuracy of the patterned plate still meets the requirements. This validates the scalability and applicability of MOARI-LC, which can effectively tap into the potential of existing equipment, and provide optimization directions for model parameter anomalies. It is of great significance for efficient production and stable transition of products.

## Figures and Tables

**Table 1:** Austenitic stainless steel HBD-SUS304 specific composition (%).

Component	Content
Cr	18.2480
Ni	8.0200
Mn	1.0100
Si	0.4200
N	0.0430
C	0.0360
Cu	0.0270
P	0.0260
Mo	0.0160
S	0.0020
Al	0
Ti	0
V	0
Nb	0
B	0

**Table 2:** Specific setting coefficients for each stand.

Specific Setting Coefficient	F1	F2	F3	F4	F5	F6
*coff*_1*i*_	1.107	0.927	0.928	0.831	0.860	0.911
*K_i_*	64,574	56,702	56,372	66,726	67,371	67,554

**Table 3:** Simulation results of each stand reduction rate (%).

Iteration	F1	F2	F3	F4	F5	F6
1	41.968	44.138	35.173	31.741	25.544	21.445
2	41.548	43.696	34.821	31.423	25.289	21.445
3	41.133	43.259	34.473	31.109	25.036	21.445
4	40.721	42.827	34.128	30.798	24.785	21.445
5	40.314	42.399	33.787	30.490	24.538	21.445
6	39.911	41.975	33.449	30.185	24.292	21.445
7	39.512	41.555	33.114	29.883	24.049	21.445
8	39.117	41.139	32.783	29.584	23.809	21.445
9	38.726	40.728	32.455	29.289	23.571	21.445
10	38.338	40.321	32.131	28.996	23.335	21.445
11	37.955	39.917	31.810	28.706	23.102	21.445
12	37.575	39.518	31.492	28.419	22.871	21.445
13	37.200	39.123	31.177	28.134	22.642	21.445
14	36.828	38.732	30.865	27.853	22.415	21.445
15	36.459	38.344	30.556	27.575	22.191	21.445
16	36.095	37.961	30.251	27.299	21.969	21.445
17	35.734	37.581	29.948	27.026	21.750	21.445
18	35.376	37.206	29.649	26.756	21.532	21.445
19	35.023	36.834	29.352	26.488	21.317	21.445
20	34.672	36.465	29.059	26.223	21.104	21.445
21	34.326	36.101	28.768	25.961	20.893	21.445
22	33.982	35.740	28.480	25.701	20.684	21.445
23	34.152	35.918	28.623	25.830	20.787	21.445

**Table 4:** Load distribution results of each stand.

Indicators	F1	F2	F3	F4	F5	F6
Entry thickness (mm)	35.803	23.575	15.108	10.783	7.998	6.335
Exit thickness (mm)	23.575	15.108	10.783	7.998	6.335	4.977
Hardness value (kN)	64,574	56,702	56,372	66,726	67,371	67,554
Stand rolling force correction factor	1.107	0.927	0.928	0.831	0.860	0.911
Load distribution (%)	75	58	46	44	37	21
Reduction rate (%)	34.152	35.918	28.623	25.830	20.787	21.445
Calculated rolling force (kN)	24,413.3	18,879.6	14,973.5	14,322.5	12,043.9	13,197.8
Actual rolling force (kN)	23,556.7	17,806.2	14,552.3	13,998.2	12,000.1	13,106.9
Percentage error (%)	3.64	6.03	2.89	2.39	0.36	0.69

**Table 5:** Comparative effects of different methods applied.

Evaluating Indicators	MOARI-LC	FCRA-NC	DCRA-GC
MAE	0.0103	0.0151	0.0235
RMSE	0.0117	0.0176	0.0274
Qualification Rate (%)	80	64	36

**Table 6:** Specific components of Type 2~Type 7 (%).

Component	Type 2	Type 3	Type 4	Type 5	Type 6	Type 7
Cr	18.1890	18.2480	0.3700	0.3520	0.0254	0.0254
Ni	8.0840	8.0200	0.1460	0.1400	0.0037	0.0037
Mn	1.1600	1.0100	0.3300	0.3700	0.3145	0.3145
Si	0.5400	0.4200	0.4300	0.4000	0.1239	0.1239
N	0.0470	0.0430	0	0	0.0035	0.0035
C	0.0490	0.0360	0.0760	0.0830	0.1576	0.1576
Cu	0.0810	0.0270	0.2650	0.2840	0.0044	0.0044
P	0.0350	0.0260	0.0750	0.0960	0.0171	0.0171
Mo	0.0930	0.0160	0	0	0.0022	0.0022
S	0.0010	0.0020	0.0020	0.0040	0.0152	0.0152
Al	0	0	0	0	0.0010	0.0010
Ti	0	0	0	0	0.0010	0.0010
V	0	0	0	0	0.0012	0.0012
Nb	0	0	0	0	0.0010	0.0010
B	0	0	0	0	0	0

**Table 7:** Prediction error of rolling force (%).

Steel Grade and Type	F1	F2	F3	F4	F5	F6
2, HBD-SUS304-1	−1.25	−5.80	−4.12	−4.87	−0.10	−0.85
3, HBD-SUS304-2	−0.52	−1.55	−2.57	4.90	1.91	0.59
4, H-09CUPCRNIA-1	10.7	7.65	2.94	1.35	−0.58	−1.48
5, H-09CUPCRNIA-2	3.21	8.83	7.10	4.63	1.01	−2.12
6, H-Q235A-1	−5.35	−7.66	−0.69	0.05	−2.13	−0.16
7, H-Q235A-2	−1.36	4.55	5.87	−3.72	−2.33	−0.79

**Table 8:** Numerical results of specific experiments.

Indicators	Type 2	Type 3	Type 4	Type 5	Type 6	Type 7
target thickness (mm)	2.91	3.88	2.50	7.90	1.80	2.90
target width (mm)	1029	1249	900	1150	1250	1250
finishing rolling target thickness (mm)	2.961	3.955	2.530	8.004	1.821	2.937
finishing rolling entry thickness (mm)	35.785	35.795	42.665	45.784	32.573	42.744
finishing rolling entry width (mm)	1055	1286	926	1185	1258	1291
the actual thickness (mm)	2.935	3.901	2.497	7.859	1.805	2.915
the pattern height (mm)	0.590	0.790	0.510	1.600	0.380	0.610
the pattern height to thickness ratio	0.201	0.203	0.204	0.204	0.211	0.209

## Data Availability

The data presented in this study are available to corresponding authors upon request. The data are not published for privacy reasons.

## Abbreviations

The following abbreviations are used in this manuscript:

NSGA-II	Non-dominated sorting genetic algorithm
MOARI-LC	Multi-objective adaptive rolling iteration method (local constraints)
FCRA-NC	Fixed compression rate allocation method (no constraints)
DCRA-GC	Dynamic compression rate allocation method (global constraints)
Pci	Calculation of rolling force of the first stand of finishing rolls
coff1i	Rolling force correction factor for stand i
Ki	Brinell hardness value of steel
εi	Reduction rate of stand i
εei	Final reduction rate of stand i
ht	Cooling value of target thickness in finishing rolling
Pmax	Maximum rolling force of each stand in finishing rolls
coff2	Steel grade correction factor
εpi	The load of stand i is scheduled to be distributed under reduction
h0i	Stand i entry thickness
hi	Stand i exit thickness
